# Operative results after xiphoidectomy in patients with xiphodynia

**DOI:** 10.1177/02184923211019179

**Published:** 2021-05-20

**Authors:** Frank P Garssen, Margot B Aalders, Marcel J van der Poel, Wietse P Zuidema

**Affiliations:** 1 Department of Surgery, Amstelland Hospital, Amstelveen, The Netherlands; 2 Department of Surgery, Amsterdam University Medical Center, Amsterdam, The Netherlands

**Keywords:** Xiphodynia, xiphoidectomy, thoracic surgery, surgical outcome, chest, sternal

## Abstract

**Background:**

Xiphodynia, the painful xiphoid process, is a rare condition with an atypical presentation. Symptoms differ in severity and site, and can consist of chest, throat, and upper abdominal pain. Primarily, other more severe causes of these symptoms need to be excluded. After this exclusion as xiphodynia is diagnosed, treatment can consist of a multitude of options, since there is no consensus regarding the optimal treatment. The aim of this study was to describe the outcomes and efficacy of one of the options, namely surgical resection of the xiphoid in patients with xiphodynia.

**Methods:**

In this retrospective case series, all consecutive patients that underwent xiphoidectomy for xiphodynia between January 2014 and December 2017 were included. Patients’ medical files including pre-operative work up, NRS scores, surgical outcomes, and follow up were reviewed. All patients received a questionnaire with follow-up questions.

**Results:**

A total of 19 patients were included. None of the patients had surgery-related complications. Response rate of the questionnaire was 84% and showed that 94% of patients had an improvement of complaints after surgery, with 10 patients (63%) being totally pain free, after a mean follow-up from 34 months after surgery.

**Conclusions:**

Xiphoidectomy is feasible and safe for the treatment of patients with xiphodynia with an improvement of complaints in nearly all patients.

## Introduction

Xiphodynia is most often described as a pain in the xiphoid process that can present as atypical chest, abdominal, back, throat, and arm pain or pain in the epigastric region.^1–15^ Typically, the pain occurs at bending or twisting movements or lifting heavy objects.^5,10^ A significant increase in pain upon direct pressure to the xiphoid is one of the most contributing findings during physical examination.^1,3,7,8,13^ Before a diagnosis can be made, it is essential that other, more severe causes of chest and epigastric pain are ruled out, especially since complaints of xiphodynia can differ widely.^1–3,7,8,13^ Normal work up is an electrocardiogram, ultrasound upper abdomen, gastroscopy, and/or computed tomography.

Literature discussing this condition is very limited. Since the first description of xiphodynia in 1712,^1^ only case reports and small case series have been described.^1–15^ The etiology of xiphodynia is still unknown, but (recurring) traumatic injury might contribute.^4^ Descriptions of treatment of xiphodynia consists of several options including physiotherapy, local anesthetic injections and surgical removal of the xiphoid (xiphoidectomy).^1,4,5,7,11^

Theoretically, xiphoidectomy offers the only permanent treatment option, since the origin of complaints is surgically removed. However, very little is known about the surgical outcomes and effectiveness of this procedure.

The aim of the current study was to describe the effect of surgical treatment in the largest to date case series of patients in which xiphoidectomy for xiphodynia was performed.

## Patients and methods

### Patients

A retrospective, single center case series was performed, including all consecutive, adult patients undergoing xiphoidectomy for xiphodynia from 1 January 2014 until 31 December 2017. There were no exclusion criteria other than the required significant increase in pain upon direct pressure to the xiphoid and exclusion of more severe causes of chest and epigastric pain. A list of patients undergoing xiphoidectomy was prospectively maintained so no patients were missed. All patients gave informed consent. The medical ethical committee of the hospital gave permission for the study.

### Data collection and study outcomes

Electronic patient files were retrospectively reviewed in order to collect study data, which were registered in a protected Excel® database. Routine follow-up was scheduled at four weeks after surgery in all patients. Additionally, all patients were contacted and received a follow-up questionnaire after 2.5 years.

Baseline characteristics included patient demographics, duration, nature and severity of preoperative symptoms, presence of recurring micro trauma to the xiphoid, additional examinations, previous consultations with other specialties and previous treatment. Severity of symptoms was measured using a Numeric Rating Scale (NRS) ranging from 0 to 10, with 0 being no pain and 10 being the most severe pain imaginable.

The primary outcome of this study was relief of symptoms, which was measured by comparing pre and postoperative NRS scores. Secondary outcomes included patient satisfaction and operative complications. For measurement of patient satisfaction, a five-point Verbal Rating Scale (VRS) was used.

### Surgical technique

**Figure 1.**
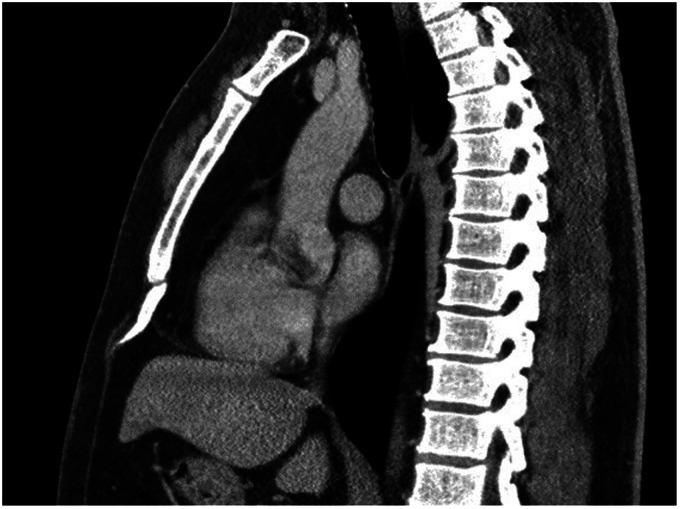
CT image (sagittal plane) showing the xiphoid process bending forwards and sticking out in the soft tissues.

**Figure 2.**
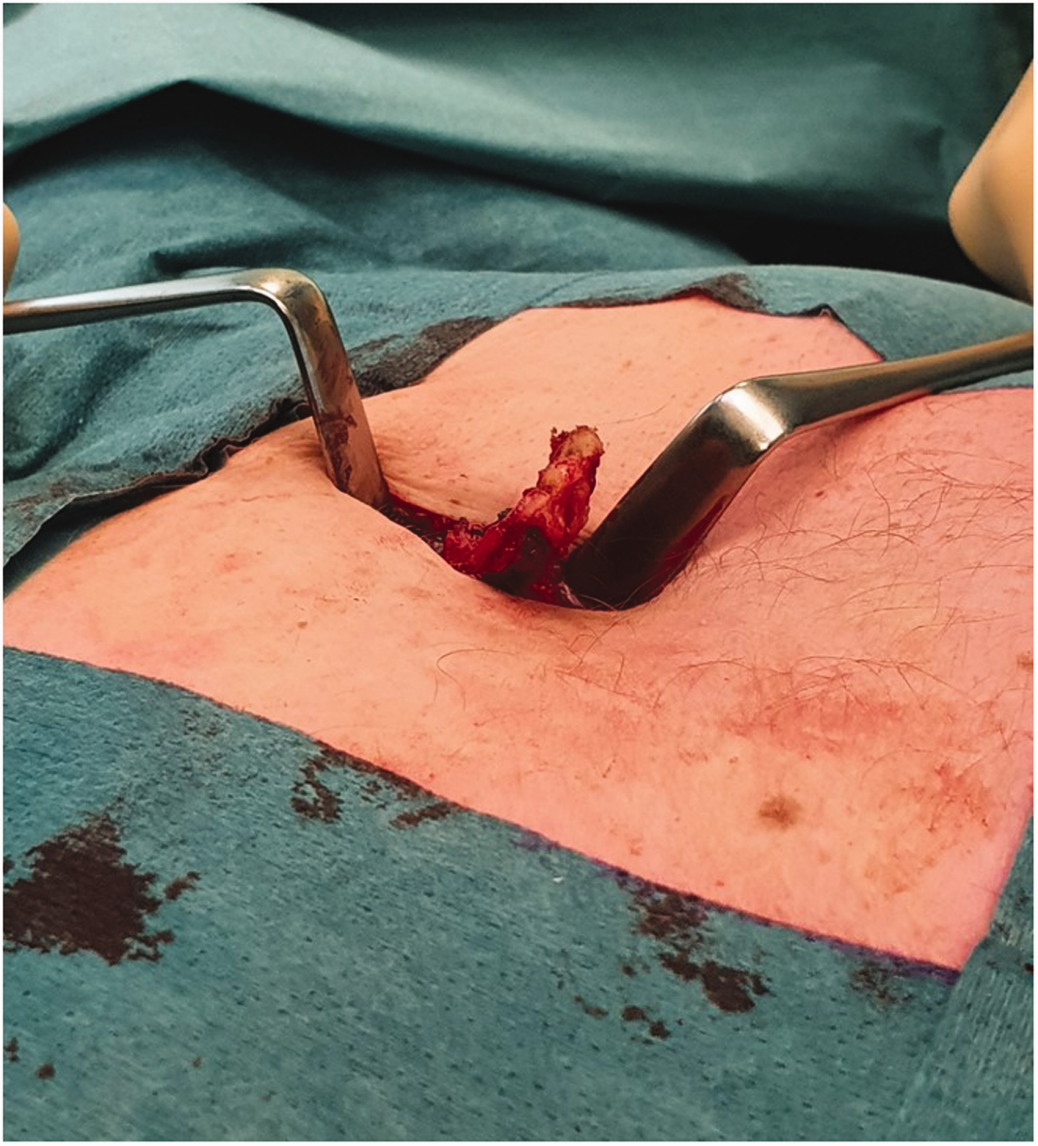
Operative image of the curved xiphoid process after being released from surrounding tissue, before removal.

Xiphoidectomy is performed with the patient in supine position under general anesthesia by making a 3-cm midline incision at xiphoid level. The xiphoid is excised with the perichondrium and/or removed with diathermy (Figures 1 and 2.) Bone nibbling forceps are used for correcting sharp edges. The abdominal fascia is closed with a PDS® loop and the skin is closed intracutaneously. Twenty milliliter of a long-acting local anesthetic (Ropivacaine® 7.5 mg/ml) is injected in the surrounding soft tissue.

## Results

### Baseline and operative results

A total of 19 patients underwent xiphoidectomy for xiphodynia during the study period. None of them had a history of surgery in the sternal area. Table 1 summarizes the preoperative patients’ characteristics. All patients were treated in day care and went home the same day. None of the patients showed any complications during or after surgery.

**Table 1.** Patient characteristics.

	Overall *n* = 19
Age, years, mean (range)	62 (33–76)
Sex	
Male	8 (42)
Female	11 (58)
Traumatic injury	
Yes	5 (26)
No	11 (58)
Unknown	3 (16)
Duration complaints, weeks, median(range)	91 (2–1300)
Earlier specialties	
None	5 (26)
Gastroenterology	9 (47)
Internal medicine	9 (47)
Cardiology	2 (11)
Other	2 (11)
Earlier examinations	
None	4 (21)
X-thorax	10 (63)
Ultrasound	14 (74)
CT-scan	6 (37)
MRI	4 (21)
Gastroscopy	8 (42)
ERCP	1 (5)
Earlier treatment	
None	5 (26)
Physiotherapy	4 (21)
Manual therapy	1 (5)
Local injections	9 (47)
Medication	
Painkillers	7 (37)
Antacids	4 (21)
Vit D	1 (5)
Pain	
Yes	18 (95)
No	1 (5)
Pressure pain xiphoid	
Yes	19 (100)
No	0

Note: All values in parentheses represent percentages, unless stated otherwise. Multiple options were possible for earlier specialties, examinations and treatments.

### Follow-up

At four weeks after surgery, 14 of the 19 (74%) patients were free of pain, 4 (21%) patients experienced a decrease of pain and 1 (5%) patient did not attend the outdoor clinic appointment.

Sixteen (84%) patients returned the questionnaire, with follow-up after surgery ranging after a mean follow-up from 34 months after surgery. Of these 16 patients, all but one experienced a reduction in complaints after surgery, while 10 patients (63%) remained free of pain (Table 2). The mean NRS score of these 16 patients before treatment was 7.7 (2–9) compared to a mean NRS score at late follow-up of 1.1 (0–5).

**Table 2.** Level of satisfaction after treatment using verbal rating scale.

	Overall*n = *16
I am very satisfied; I never experience pain	10 (63)
I am very satisfied; I occasionally experience some pain	2 (13)
I have improved but experience pain on a regular basis	3 (19)
I have had no result on this treatment	1 (6)
My pain is worse after treatment	0

Note: All values in parentheses represent percentages.

## Discussion

Nearly all patients suffering from xiphodynia in the current study experienced a relieve of complaints after xiphoidectomy without any surgical complications, suggesting that surgical removal of the xiphoid process is an effective treatment strategy in these patients.

Very little is known about xiphodynia and current literature is scarce. According to Lipkin et al.,^2^ the first xiphoidectomy was reported in 1852 and since then only case reports and small case series have been published, leaving much to be debated.^1–15^ The number of patients included in this study in a relative short period of time (4 years) is probably caused more by the dense population of the Netherlands and the open health care system than by a higher incidence of xiphodynia in the Dutch population. Remarkable is the loss of quality of life in these group patients, caused mainly by intermitted pain and urge to avoid the trigger of the pain.

It is hypothesized that recurrent traumatic injury, recent weight loss, previous pregnancy or previous surgery of the xiphoid process may contribute to the development of xiphodynia.^3,4^ In our study group, one patient had complaints fitting xiphodynia started two months after given birth; however, these complaints spontaneously disappeared within months, but relapsed after 4.5 years. A relationship between loss of weight and occurring of the complaints could not be established in this study group. This series shows five persons had a history of trauma to the chest wall, but only one person had a sternal fracture at the xiphisternal joint. Maigne et al. proposed that mechanical discomfort and local inflammation caused by an abnormal anterior prominence of the xiphoid might be of influence.^10^ In the patients, the resected specimens were reviewed and showed no inflammatory or other abnormalities.

Patients mostly present with long-lasting pain in epigastric, often radiating to the arms, neck, and back. In a majority of the patients, the complaints are intermittent and are provoked by certain movements, like bending down, eating large meals or exertion. The pain on clinical examination is located directly over the xiphoid and provoked by direct pressure. One could argue that by applying downforce pressure over the xiphoid, the exact location of the pain could be confined to the xiphoid but could also be located in the sternal-xiphoid junction, although never established. The complaints can mimic more life-threatening diseases, which need to be excluded first. This is according to the current practice in our center. For example, the radiating pain in an arm can be a sign of cardiac illness, likewise radiating pain in the back can be a sign of vascular (aortic) disease or neurological disease. Pain in the epigastric region can be caused by stomach, esophagus disorders or abdominal wall hernia. The provocation of pain by certain movements suggests a partly mechanical issue. The majority of patients treated have seen several specialists and underwent several additional examinations to exclude other diseases. Scintigraphy was not added to the workup, because from the study of Siberstein et al., we know that the natural uptake is increased in xiphoid cartilage, tendon, and ear cartilage, which can lead to improper conclusions. Scintigraphy can however be useful in the diagnosis of sternal fractures during the early period after thorax trauma (Erhan et al.) or for diagnosis of deep sternal infections after sternotomy for cardiac illnesses (Liberatore et al.).^16–18^ We stress the importance of excluding any life-threatening diseases first, before diagnosing a patient with xiphodynia.

A useful addition to strengthen the decision making process could be the possible disappearance of pain after local injection over the xiphoid with bupivacaine®. Once diagnosed, the proposed treatment options for xiphodynia vary from physiotherapy and oral use or local injection of analgesics to surgical removal of the xiphoid. However, the optimal treatment strategy has not yet been identified.^6,7,15^

In recent literature, xiphoidectomy is described several times as treatment for xiphodynia, with mostly successful results.^1,3,6,8,15^ Theoretically, xiphoidectomy offers a permanent solution to the complaints but can be associated with the inherent risk of complications of a surgical intervention. First of all, the current study showed that xiphoidectomy offers at least a partial relieve of symptoms in nearly all patients but still a few patients experienced residual pain. Only 63% of patients were pain free after follow-up. These results suggest that xiphoidectomy is indeed an effective treatment strategy. A difference in treatment effect on specific patients may be expected based on their preoperative baseline characteristics. Dom et al. proposed that patients suffering from xiphodynia who had undergone previous surgery in the epigastric area might not benefit from xiphoidectomy, while patients with pressure pain and an abnormal ventral protrusion of the xiphoid might.^3^ The current study did not identify new predictors of treatment effect. It is speculated that patients that experience a clear relieve of symptoms after local injection with a long acting analgesic might be the best responders to surgery.

Furthermore, none of the patients in the current study or in the scarce literature experienced any complications during surgery or follow-up, clearly demonstrating the safety and minimally invasiveness of xiphoidectomy.

This study has several limitations. First, although it is a large case series about this relative rare condition, the number of patients is small and the nature of the study is retrospective, making it necessary to interpret conclusions carefully. Second, little is known about the underlying pathophysiology which again is a cause for caution. Third are the unknown causal relationships of the condition with other physical or psychological factors, making any correlation sensitive to bias.

In conclusion, before diagnosing xiphodynia, other, more severe diseases must be excluded. Once diagnosed, xiphoidectomy is likely to offer a relieve of symptoms with very little risk of postoperative complications.
